# The Association Between Obesity and Depressive Symptoms: Mediation by C-Reactive Protein and Neutrophil-to-Lymphocyte Ratio

**DOI:** 10.31083/AP45975

**Published:** 2025-08-22

**Authors:** Pu-Le Liu, Yan Zhang, Jiao Li, Jing Du, Ning Yang, Qiang-Li Dong

**Affiliations:** ^1^Department of Mental Health, The Second Hospital of Lanzhou University, 730000 Lanzhou, Gansu, China; ^2^Department of Mental Health, Mental Health Institute of Central South University, 410000 Changsha, Hunan, China

**Keywords:** depressive symptom, inflammatory mediators, obesity, CRP, NLR, NHANES

## Abstract

**Background:**

Obesity and depressive disorders are significant public health concerns, and their association is well-documented. This study investigates the role of inflammatory markers, specifically C-reactive protein (CRP) and neutrophil-to-lymphocyte ratio (NLR), in mediating the relationship between obesity and depressive symptoms.

**Methods:**

We utilized data from 37,538 adults from the National Health and Nutrition Examination Survey (NHANES), covering the period from 2005 to March, 2020, pre-pandemic. Depressive symptoms were measured using the Patient Health Questionnaire-9 (PHQ-9), while inflammatory markers were assessed via NLR and CRP levels.

**Results:**

Results indicated a positive correlation between obesity, NLR, and CRP levels, and depressive symptoms. Notably, CRP exhibited a significant mediating effect in the obesity and depressive symptoms link, whereas NLR did not. (NLR: 0.0926%, *p* = 0.740; CRP: 32%, *p* < 0.001). Furthermore, the mediating effect of CRP in the male group was significantly higher than in the female group (Men: 57%, *p* < 0.001; Women: 16%, *p* = 0.046).

**Conclusion:**

These findings provide new insights into the mechanisms linking obesity and depressive symptoms, especially in men, and may guide future therapeutic strategies.

## Main Points

- ** Obesity and Depressive Symptoms**: A significant association was found between obesity and depressive symptoms, which was stronger in women.
- ** Inflammatory Markers (CRP and NLR)**: Obesity was linked to higher levels of C-reactive protein (CRP) and neutrophil-to-lymphocyte ratio (NLR), both of which are associated with depressive symptoms.
- ** Mediation by CRP**: CRP played a key mediating role between obesity and depressive symptoms, with a stronger effect in men.
- ** No Significant Mediation by NLR**: While NLR was associated with both obesity and depressive symptoms, it did not show significant mediation between them.
- ** Future Research and Treatment**: Future longitudinal research to explore causal relationships and personalized treatments based on inflammatory markers is suggested.

## 1. Introduction

Obesity and depressive disorders are prevalent conditions with substantial public health implications. They often co-occur, leading to significant morbidity. Previous research has shown a bidirectional relationship between obesity and depressive disorders, with one increasing the risk of the other. The association between obesity and depressive symptoms is well established, with evidence suggesting a bidirectional interaction between the two [1]. Recent systematic reviews and meta-analyses further emphasize this relationship, reporting that the prevalence of obesity among individuals with major depressive disorder (MDD) ranges from 10.1% to 26.7%, suggesting that obesity significantly contributes to the development of depressive symptoms, particularly in high-risk populations such as children, adolescents, and women [2]. The study has observed varying obesity–depression associations across different racial and ethnic groups, highlighting the importance of considering these differences when exploring potential interventions [3]. The relationship between obesity and depression is thought to be mediated by inflammation. Both human and animal study has shown that individuals with obesity often have elevated levels of inflammatory markers [4]. Obesity is commonly associated with a mild, chronic systemic inflammation, as evidenced by elevated levels of inflammatory markers in individuals who are obese [5]. Adipose tissue plays a significant role as an endocrine organ, secreting pro-inflammatory adipokines such as leptin and tumor necrosis factor-alpha (TNF-α), which play a part in promoting systemic inflammation [6]. The increase in inflammation levels, in turn, exacerbates obesity [7]. Meanwhile, this inflammatory environment can extend to where inflammatory markers may cross the blood-brain barrier, activating microglial cells and potentially contributing to neuroinflammation, which has been associated with the onset of depressive symptoms [8]. Recent research suggests that neuroinflammation, specific activation of the immune system in the central nervous system, may play a crucial role in altering mood and behavior in individuals with obesity [9]. Inflammatory markers, therefore, hold potential as biomarkers for assessing depressive symptoms, reflecting both the disease state and response to treatment [10]. Additionally, anti-inflammatory treatments have been shown to alleviate depressive symptoms to varying degrees [11].

Furthermore, sex differences in the obesity–depression relationship well documented, with women generally being at higher risk for depression than men. These differences are thought to be influenced by hormonal factors, as well as social and behavioral factors. For instance, inflammation and its effects on depression appear to be higher in women, potentially due to differences in fat distribution and immune system functioning between sexes [12]. Additionally, disparities in inflammatory responses in the obesity–depression relationship between different racial groups have been noted, potentially contributing to varying outcomes among different ethnic groups [13].

Although previous studies have shown a relationship between obesity and depressive symptoms, the specific mechanisms through which inflammatory markers mediate this relationship and which markers play the most significant role remain unclear. This knowledge gap has prompted us to explore the mediating role of two inflammatory markers—C-reactive protein (CRP) and neutrophil-to-lymphocyte ratio (NLR)—in this context. Both CRP and NLR are key indicators of systemic inflammation, which plays a crucial role in the pathway linking obesity and depression. CRP, a well-known acute-phase protein, is elevated during inflammatory responses and has been directly associated with the pro-inflammatory state observed in both obesity and depression [1]. On the other hand, NLR, derived from routine blood tests, reflects the balance between neutrophils and lymphocytes and is indicative of ongoing inflammation and stress, factors that have been linked to both obesity and depressive symptoms [14]. While other inflammatory markers, such as IL-6 and chemokines, are also important, CRP and NLR were chosen for this study due to their greater clinical accessibility, cost-effectiveness, and well-documented associations with both obesity and depression. These markers provide a solid foundation for investigating their mediating effects in the relationship between obesity and depressive symptoms.

Previous systematic reviews suggest that sex hormones influence the relationship between obesity and depressive symptoms, with women being at higher risk of developing depression [12]. Considering these potential sex differences, we examined the associations between inflammation, obesity, and depressive symptoms separately for men and women. Given this, we hypothesized that inflammatory markers such as CRP and NLR play a different mediating role in the relationship between obesity and depression in women compared with men.

In our research, which analyzes data from the National Health and Nutrition Examination Survey (NHANES) from 2005 to March, 2020, pre-pandemic (n = 76,496), we also explored these relationships across diverse racial groups.

This study aimed to address three main questions: (1) Is there a connection between obesity, CRP, and NLR, and depressive symptoms? (2) Do CRP and NLR act as mediators between obesity and depressive symptoms? (3) Do sex differences influence the mediating effects of CRP and NLR in this relationship? Our hypotheses were: (1) There is a positive correlation between obesity, CRP, and NLR, and depressive symptoms; (2) CRP and NLR may partly mediate the effect of obesity on depressive symptoms; (3) Sex differences may influence the mediating role of CRP and NLR in this relationship.

## 2. Materials and Methods

### 2.1 Study Population

We performed a cross-sectional analysis utilizing data from the NHANES program, managed by the Centers for Disease Control and Prevention (CDC), covering the period from 2005 to March, 2020, pre-pandemic. The NHANES survey is designed to evaluate the health and nutritional conditions of the US population. The study protocol was approved by the Ethics Review Board of the National Center for Health Statistics (NCHS), ensuring compliance with the ethical standards outlined in the updated Declaration of Helsinki. All participants gave written informed consent before being included in the study. For more detailed information about the NHANES program, please refer to the CDC website, https://www.cdc.gov/nchs/nhanes/?CDC_AAref_Val=https://www.cdc.gov/nchs/nhanes/index.htm (Centers for Disease Control and Prevention, 2024).

Participants were chosen based on these inclusion criteria: (1) aged 18 years or older, and (2) having at least one available inflammatory marker (NLR or CRP) for analysis. The exclusion criteria included: (1) incomplete Patient Health Questionnaire-9 (PHQ-9) data, (2) missing body mass index (BMI) information, and (3) reported use of anti-infective drugs, immunosuppressants, or immunostimulants.

### 2.2 Primary Measures

Depressive symptom assessment: Depressive symptoms were assessed using the nine-item PHQ-9. The scale demonstrated good reliability in the original study (Cronbach’s α = 0.89) [15]. The reliability coefficient, computed using data from the present study sample, was α = 0.87, demonstrating a strong internal consistency within the population under investigation. The PHQ-9 is widely recognized as a reliable and valid tool for screening depressive symptoms [16]. The PHQ-9 total score can vary between 0 and 27, with higher scores reflecting an increased intensity of depressive symptoms [17]. A threshold score of 10 or above has been found to maximize both sensitivity and specificity in various populations [17].

BMI: BMI is a measurement derived from a person’s weight and height, typically used to classify individuals into categories such as underweight, normal weight, overweight, and obese. It is calculated by dividing the weight (in kilograms) by the square of the height (in meters). A BMI value of 30 or higher is considered indicative of obesity [18].

To simplify the analysis and improve interpretability, participants were categorized into four groups based on PHQ-9 scores and BMI: “Depression with Obesity”, “Obesity without Depression”, “Depression without Obesity”, and “Neither Depression nor Obesity”. A PHQ-9 score greater than 9 was used to define depression, as this threshold has been widely validated in epidemiological studies as indicative of clinically significant depressive symptoms. This classification simplifies the continuous variables of the PHQ-9 scores and BMI, enabling a more straightforward examination of the co-occurrence and interactions between obesity and depressive symptoms in large populations. While we acknowledge that this approach may overlook more nuanced variations within these continuous measures, it provides a clear and interpretable framework for understanding the broad relationship between obesity and depression. Future studies could benefit from more refined analyses, such as examining the full distribution of PHQ-9 and BMI scores, to capture subtler associations between these variables.

### 2.3 Laboratory Measures

The neutrophil-to-lymphocyte ratio (NLR) is calculated by dividing the neutrophil count by the lymphocyte count. Neutrophil and lymphocyte counts, expressed as cells per liter (10^9^), were analyzed using the Beckman Coulter MAXM instrument (Beckman Coulter, Inc., Brea, CA, USA).

NLR was selected as an inflammation marker due to its ability to reflect systemic inflammation through a simple and inexpensive calculation derived from routine blood counts. Elevated NLR has been associated with various inflammatory diseases and has gained attention as a potential marker for assessing low-grade chronic inflammation in obesity [19]. The study has demonstrated that an elevated NLR is linked to both obesity and depressive symptoms, suggesting that it may serve as a useful tool for exploring the inflammatory mechanisms underlying these conditions [20]. NLR has been proposed as a reliable marker in large cohort studies due to its simplicity and cost-effectiveness, further supporting its use in this investigation.

C-reactive protein (CRP) levels were measured using different methods depending on the NHANES cycle. In the 2005–2010 cycle, CRP levels were measured using the latex-enhanced rate nephelometry method on Behring instruments (Dade Behring Diagnostics, Inc., Newark, DE, USA), with a lower limit of detection (LLOD) of 0.02 mg/dL. During the 2015 to March, 2020, pre-pandemic period, CRP levels were measured using Beckman UniCel analyzers (Beckman Coulter, Inc.), with LLOD values of 0.011 mg/dL for 2015–2016 and 0.015 mg/dL for 2017–March, 2020, pre-pandemic. For values below the LLOD, the LLOD was divided by the square root of 2 (LLOD/$\sqrt{2}$) and used as a substitute. In the NHANES cycles, different instruments were used to measure CRP levels. Specifically, the Behring instruments were used in the 2005–2010 cycle, while the Beckman UniCel analyzers were used from 2015 to March, 2020. These differences in instruments could potentially introduce bias due to variations in sensitivity and detection limits. To mitigate this potential bias, the CRP values obtained using different instruments were standardized where possible. During the analysis, values below the LLOD were substituted by dividing the LLOD by the square root of 2, a widely used approach to account for these discrepancies. Additionally, we employed statistical adjustments for potential instrument-related biases in our models.

CRP was chosen as an inflammation marker due to its well-established role as an acute-phase reactant. CRP levels rise in response to systemic inflammation, making it an ideal marker for evaluating inflammatory states associated with obesity and depression. Elevated CRP has been linked to both obesity and depressive symptoms in numerous studies, highlighting its potential as a biomarker for these conditions [9]. Recent research has shown that CRP is significantly elevated in individuals with obesity, and elevated levels of CRP have been associated with an increased risk of depression [1]. These findings support CRP’s utility in studies examining the relationship between inflammation, obesity, and depression.

### 2.4 Covariates

Continuous covariates included age. Categorical variables, used for classification, encompassed race/ethnicity (Mexican American, other Hispanic, non-Hispanic White, non-Hispanic Black, or other races), educational attainment (less than high school, high school graduate, more than high school), marital status (married/living with a partner, or never married/widowed/divorced/separated), alcohol consumption (yes/no), smoking status (never, former, current), physical activity (inactive, moderate, vigorous, or both moderate and vigorous), and the presence of comorbid conditions (yes/no).

Alcohol consumption was evaluated using two 24-hour dietary recalls, with participants categorized as alcohol consumers if they reported drinking in at least one of the recalls. Smoking status was classified as never smoked (fewer than 100 cigarettes), former smoker (smoked ≥100 cigarettes in the past but no longer smoking), or current smoker (smoked ≥100 cigarettes and currently smoking either daily or occasionally). Physical activity was assessed based on self-reported participation in vigorous activities (e.g., running or basketball) and moderate activities (e.g., brisk walking, swimming, or regular-paced cycling). Participants were considered to have comorbid conditions if they reported at least one of the following medical conditions: diabetes, kidney failure, kidney stones, heart failure, stroke, liver disease, rheumatoid arthritis, or cancer [21]. Missing data were imputed using the MissForest R package, a method leveraging random forests. This approach is particularly effective for high-dimensional datasets with both categorical and continuous predictors, offering significant computational efficiency [22].

### 2.5 Statistical Analysis

All statistical analyses were conducted using R version 4.3.1 (R Foundation for Statistical Computing, Vienna, Austria) (https://cran.r-project.org/) and employed the following related packages for analyzing interaction effects and mediation: “foreign”, “dplyr”, “magrittr”, “tidyr”, “compareGroups”, “mediation”, “lpSolve”, “missforest”, and “broom” (https://cran.r-project.org/web/packages/index.html). For all analyses, two-tailed *p*-values < 0.05 were considered statistically significant. Effect sizes (Cohen’s d for pairwise comparisons and partial η^2^ for regression-based models) were calculated to assess the practical significance of the associations and mediation effects. Cohen’s d values were interpreted as small (0.2), medium (0.5), or large (0.8), while partial η^2^ values were used to evaluate the proportion of variance explained by the predictors, with small (0.01), medium (0.06), and large (0.14) effects considered significant. Continuous variables were reported either as means with standard deviations or as medians with interquartile ranges, based on their distribution. Categorical variables were summarized in terms of frequency and percentages. Categorical variables, including sex, race/ethnicity, education level, marital status, alcohol consumption, smoking status, physical activity, and presence of comorbid conditions, were compared across the groups (e.g., “Depression with obesity”, “Obesity without depression”, “Depression without obesity”, and “Without obesity and depression”) using the chi-squared (χ^2^) test. This test was employed to evaluate the distribution of categorical variables and examine their relationship with various groups categorized by obesity and depressive symptoms. For continuous variables, data that followed a normal distribution were analyzed using one-way analysis of variance (ANOVA), while the Kruskal-Wallis H test was used for data that did not meet the assumption of normality. Multivariate logistic regression analysis was performed to assess the relationships between inflammatory markers, obesity, and depressive symptoms. To explore the moderating role of sex, the strength of the associations between depressive symptoms, obesity, and inflammatory markers was calculated separately for male and female subgroups. Mediation analysis was conducted to examine whether inflammatory markers mediated the relationship between the independent variable (obesity) and the dependent variable (depressive symptoms). A total of 1000 bootstrap iterations were performed, with the results detailing the magnitude of indirect effects, the proportion of mediation, and the associated *p*-values.

## 3. Results

### 3.1 Participant Characteristics

**Fig. 1.**
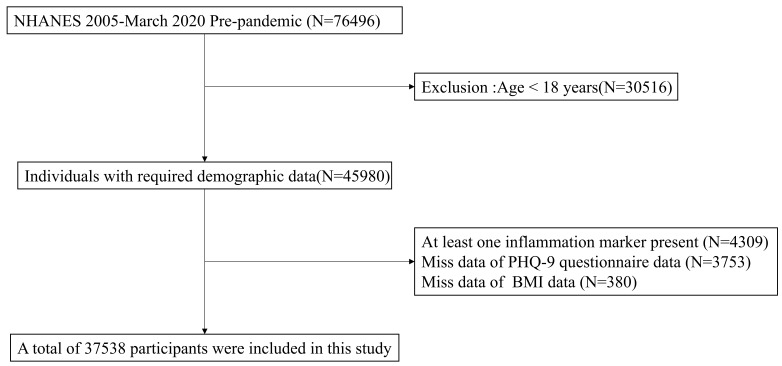
**Participant selection process**. NHANES, National Health and Nutrition Examination Survey; PHQ-9, Patient Health Questionnaire-9; BMI, body mass index.

As shown in Table 1 and Fig. 1, the study included a total of 76,496 participants. Following the screening process, a total of 37,538 participants met the inclusion criteria, which required them to be 18 years or older (n = 45,980), have available data for at least one inflammatory marker (n = 41,671), have complete PHQ-9 data (n = 37,918), and have BMI information (n = 37,538). Participants were categorized into four groups: “Depression with obesity”, “Obesity without depression”, “Depression without obesity”, and “Without obesity and depression”. A PHQ-9 score greater than 9 was used to indicate depression.

**Table 1.** **Characteristics of study participants (n = 37,538)**.

Characteristic		Total Participants	Depression with Obesity (I)	Depression without Obesity (II)	Obesity without Depression (III)	Without Obesity and Depression (IV)	*p*-value
n		37,538	1610	1673	12,704	21,551	
Age y, mean (SD)		47.9 (18.6)	49.1 (15.8)	46.3 (18.1)	49.2 (17.3)	47.2 (19.5)	<0.001
Sex n (%):							<0.001
	Male	18,455 (49.2%)	503 (31.2%)	695 (41.5%)	5850 (46.0%)	11,407 (52.9%)	
	Female	19,083 (50.8%)	1107 (68.8%)	978 (58.5%)	6854 (54.0%)	10,144 (47.1%)	
Education n (%):							<0.001
	<High school	8295 (23.4%)	510 (32.5%)	564 (36.1%)	2824 (23.0%)	4397 (21.9%)	
	Completed high school	8208 (23.2%)	389 (24.8%)	389 (24.9%)	2987 (24.3%)	4443 (22.2%)	
	>High school	18,941 (53.4%)	670 (42.7%)	608 (38.9%)	6462 (52.7%)	11,201 (55.9%)	
Race n (%):							<0.001
	Mexican American	5924 (15.8%)	265 (16.5%)	239 (14.3%)	2242 (17.6%)	3178 (14.7%)	
	Other Hispanic	3669 (9.77%)	213 (13.2%)	226 (13.5%)	1196 (9.41%)	2034 (9.44%)	
	Non-Hispanic White	15,720 (41.9%)	640 (39.8%)	690 (41.2%)	5047 (39.7%)	9343 (43.4%)	
	Non-Hispanic Black	8220 (21.9%)	391 (24.3%)	358 (21.4%)	3472 (27.3%)	3999 (18.6%)	
	Other Race	4005 (10.7%)	101 (6.27%)	160 (9.56%)	747 (5.88%)	2997 (13.9%)	
Marital n (%):							<0.001
	Married/Living with partner	21,270 (59.2%)	742 (47.1%)	695 (43.9%)	7511 (60.6%)	12,322 (60.4%)	
	Widowed/Divorced/Separated/Never married	14,663 (40.8%)	832 (52.9%)	888 (56.1%)	4877 (39.4%)	8066 (39.6%)	
Alcohol n (%):							<0.001
	No	31,647 (90.0%)	1436 (94.7%)	1401 (89.9%)	11,193 (92.8%)	17,617 (88.0%)	
	Yes	3516 (10.00%)	80 (5.28%)	157 (10.1%)	868 (7.20%)	2411 (12.0%)	
Smoke n (%):							<0.001
	Never	20,314 (56.0%)	711 (44.6%)	636 (39.5%)	7201 (57.9%)	11,766 (57.0%)	
	Former	8688 (23.9%)	412 (25.8%)	303 (18.8%)	3272 (26.3%)	4701 (22.8%)	
	Current	7296 (20.1%)	471 (29.5%)	671 (41.7%)	1974 (15.9%)	4180 (20.2%)	
Exercise level n (%):							<0.001
	Inactive	18,903 (52.7%)	915 (59.4%)	852 (53.9%)	6273 (51.6%)	10,863 (52.7%)	
	Moderate	8563 (23.9%)	305 (19.8%)	369 (23.4%)	3003 (24.7%)	4886 (23.7%)	
	Vigorous	1773 (4.94%)	87 (5.65%)	71 (4.49%)	563 (4.63%)	1052 (5.10%)	
	Both moderate and vigorous	6660 (18.6%)	234 (15.2%)	288 (18.2%)	2319 (19.1%)	3819 (18.5%)	
Comorbidity n (%):							<0.001
	No	25,599 (68.2%)	733 (45.5%)	1027 (61.4%)	7749 (61.0%)	16,090 (74.7%)	
	Yes	11,939 (31.8%)	877 (54.5%)	646 (38.6%)	4955 (39.0%)	5461 (25.3%)	
	PHQ-9 score, mean (SD)	2.59 (3.66)	12.1 (3.75)	11.8 (3.58)	1.91 (2.14)	1.56 (1.98)	0.000
	NLR, median (Q1–Q3)	1.94 (1.44–2.58)	2.07 (1.50–2.79)	1.95 (1.43–2.66)	1.96 (1.47–2.59)	1.90 (1.42–2.55)	<0.001
	CRP mg/dL, median (Q1–Q3)	1.91(0.80–4.56)	4.54 (2.30–8.91)	1.40 (0.60–3.70)	3.50 (1.70–7.09)	1.20 (0.51–2.81)	<0.001

NLR, neutrophil-to-lymphocyte ratio; CRP, C-reactive protein.

The average age of participants was 47.9 years, with 49.2% being male and 50.8% female. Racial/ethnic distribution was as follows: 15.8% Mexican American, 9.77% other Hispanic, 41.9% non-Hispanic White, 21.9% non-Hispanic Black, and 10.7% from other racial groups. The prevalence data indicated that 8.74% of the population had depressive symptoms, while 38.13% were classified as obese. Among those with depressive symptoms, 49.04% also had obesity, and 11.24% of the obese population had depressive symptoms. Median CRP levels and NLR were 4.20 mg/dL and 2.17, respectively.

Analysis of inflammatory markers revealed significant differences in NLR and CRP levels across the four groups (“Depression with obesity”, “Obesity without depression”, “Depression without obesity”, and “Without obesity and depression”) (*p* < 0.001).

### 3.2 Correlations Between Obesity, Depressive Symptoms, and NLR and CRP Levels

**Fig. 2.**
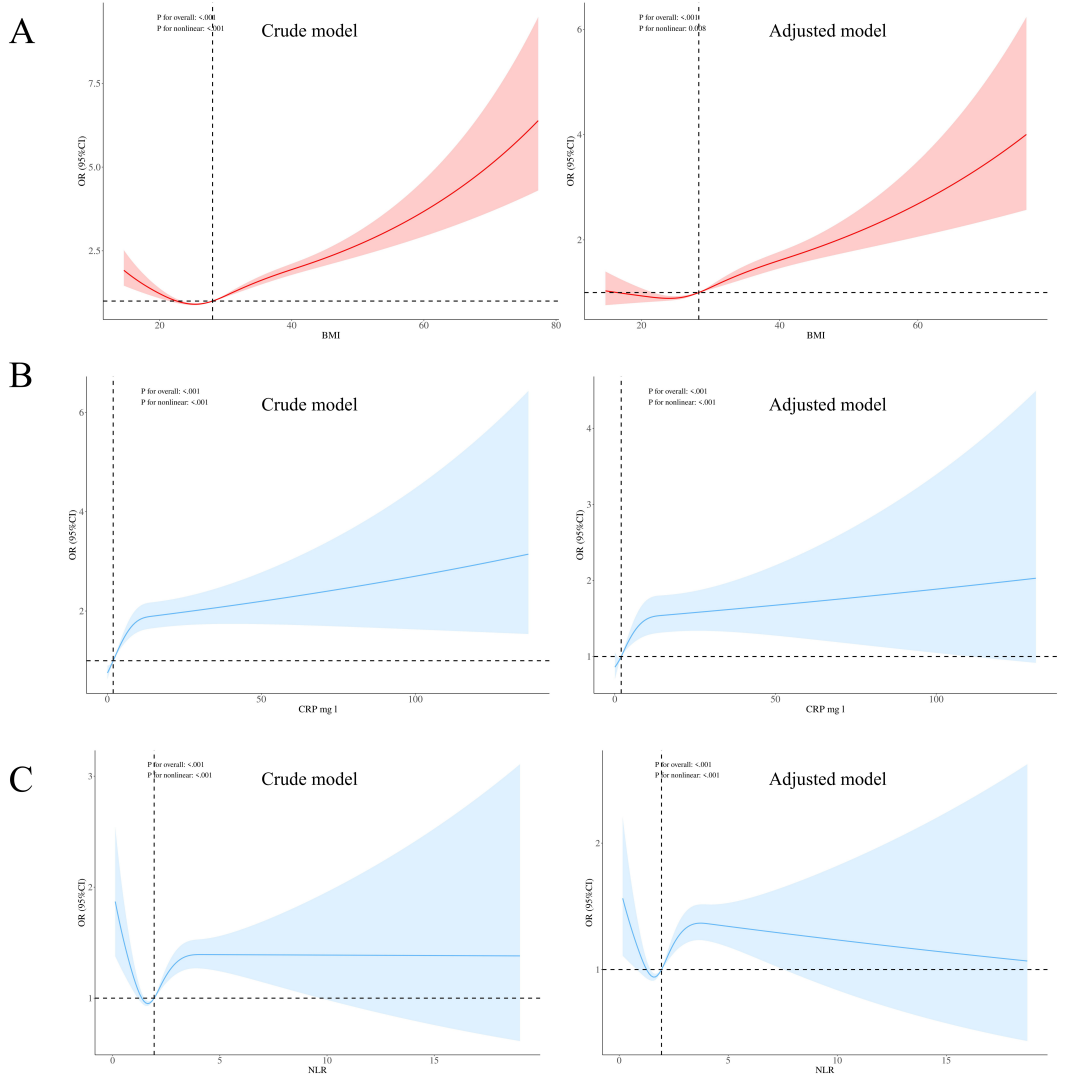
**Correlations between Obesity, Depressive Symptoms, and NLR and CRP Levels**. (A) The association between BMI and depressive symptoms. (B) The association between CRP levels and depressive symptoms. (C) The association between NLR and depressive symptoms. CRP is expressed in mg/dL and NLR is calculated as the ratio of neutrophil count to lymphocyte count. Both CRP and NLR were transformed using natural logarithms for the analysis. Crude models were not adjusted, while adjusted models accounted for variables such as age, sex, race, alcohol consumption, smoking status, education, marital status, exercise level, and comorbidities.

As shown in Table 2 and Fig. 2, the unadjusted model revealed significant positive correlations between obesity, depressive symptoms, and NLR and CRP levels. However, after adjusting for confounding markers such as age, sex, race, alcohol consumption, smoking status, education level, marital status, exercise level, and presence of a comorbidity, the association between obesity and NLR was no longer significant (NLR: β = 0.001737, 95% CI = –0.0089–0.0123, *p* = 0.748, Cohen’s d = 0.10; CRP: β = 0.922152, 95% CI = 0.8911–0.9532, *p* < 0.01, Partial η^2^ = 0.05). Sex was found to moderate the relationships between obesity, depressive symptoms, and NLR and CRP levels. Additionally, both NLR and CRP levels were positively correlated with depressive symptoms (NLR: β = 0.014480, 95% CI = 0.0078–0.0211, *p* < 0.01, Cohen’s d = 0.30; CRP: β = 0.009853, 95% CI = 0.0070–0.0128, *p* < 0.01, Cohen’s d = 0.28). Due to the large sample size, statistically significant differences were observed in many analyses. It is important to note that statistical significance does not always indicate substantial practical differences. Therefore, effect size measures are provided to give context to the significance of the findings.

**Table 2.** **Associations between obesity, inflammatory markers, and depression (n = 37,538)**.

Variable		Crude β (95% CI)	*p*	Adjusted β (95% CI)	*p*	Effect Size (Cohen’s d/Partial η^2^)
Association of obesity with depression						
	Total	0.040440 (0.0346, 0.0463)	<0.001	0.031806 (0.0254, 0.0382)	<0.001	Cohen’s d = 0.23
	Male	0.021747 (0.0143, 0.0292)	<0.001	0.019103 (0.011, 0.0273)	<0.001	Cohen’s d = 0.28
	Female	0.051119 (0.0422, 0.0601)	<0.001	0.041151 (0.0314, 0.0509)	<0.001	Cohen’s d = 0.29
Association of obesity with NLR						
	Total	0.022040 (0.0122, 0.0319)	<0.001	0.001737 (–0.0089, 0.0123)	0.748	Cohen’s d = 0.10
	Male	0.030345 (0.0157, 0.0449)	<0.001	0.005281 (–0.0102, 0.0208)	0.504	Cohen’s d = 0.15
	Female	0.018271 (0.0050, 0.0316)	<0.001	0.005345 (–0.0091, 0.0198)	0.467	Cohen’s d = 0.08
Association of obesity with CRP						
	Total	1.020641 (0.9916, 1.0497)	<0.001	0.922152 (0.8911, 0.9532)	<0.001	Partial η^2^ = 0.05
	Male	0.82064 (0.7790, 0.8630)	<0.001	0.737754 (0.6930, 0.7830)	<0.001	Partial η^2^ = 0.06
	Female	1.16352 (1.1240, 1.2030)	<0.001	1.097530 (1.0550, 1.1400)	<0.001	Partial η^2^ = 0.04
Association of NLR with depression						
	Total	0.01254 (0.0065, 0.0186)	<0.001	0.014480 (0.0078, 0.0211)	<0.001	Cohen’s d = 0.30
	Male	0.019183 (0.0118, 0.0266)	<0.001	0.017340 (0.0090, 0.0257)	<0.001	Cohen’s d = 0.35
	Female	0.00797 (–0.0016, 0.0176)	0.104	0.011250 (0.0009, 0.0216)	0.033	Cohen’s d = 0.28
Association of CRP with depression						
	Total	0.014478 (0.01192, 0.01704)	<0.001	0.009853 (0.0070, 0.0128)	<0.001	Cohen’s d = 0.28
	Male	0.011638 (0.0083, 0.0150)	<0.001	0.010400 (0.0066, 0.0142)	<0.001	Cohen’s d = 0.30
	Female	0.013437 (0.0095, 0.0173)	<0.001	0.008693 (0.0044, 0.0130)	<0.001	Cohen’s d = 0.26

CI, confidence interval. Model1, Crude. Model2, Adjusted: Age, Sex, Race, Alcohol, Smoke, Education, Marital, Exercise level, Comorbid.

### 3.3 Mediating Role of NLR and CRP Level in the Relationship Between Obesity and Depressive Symptoms 

**Fig. 3.**
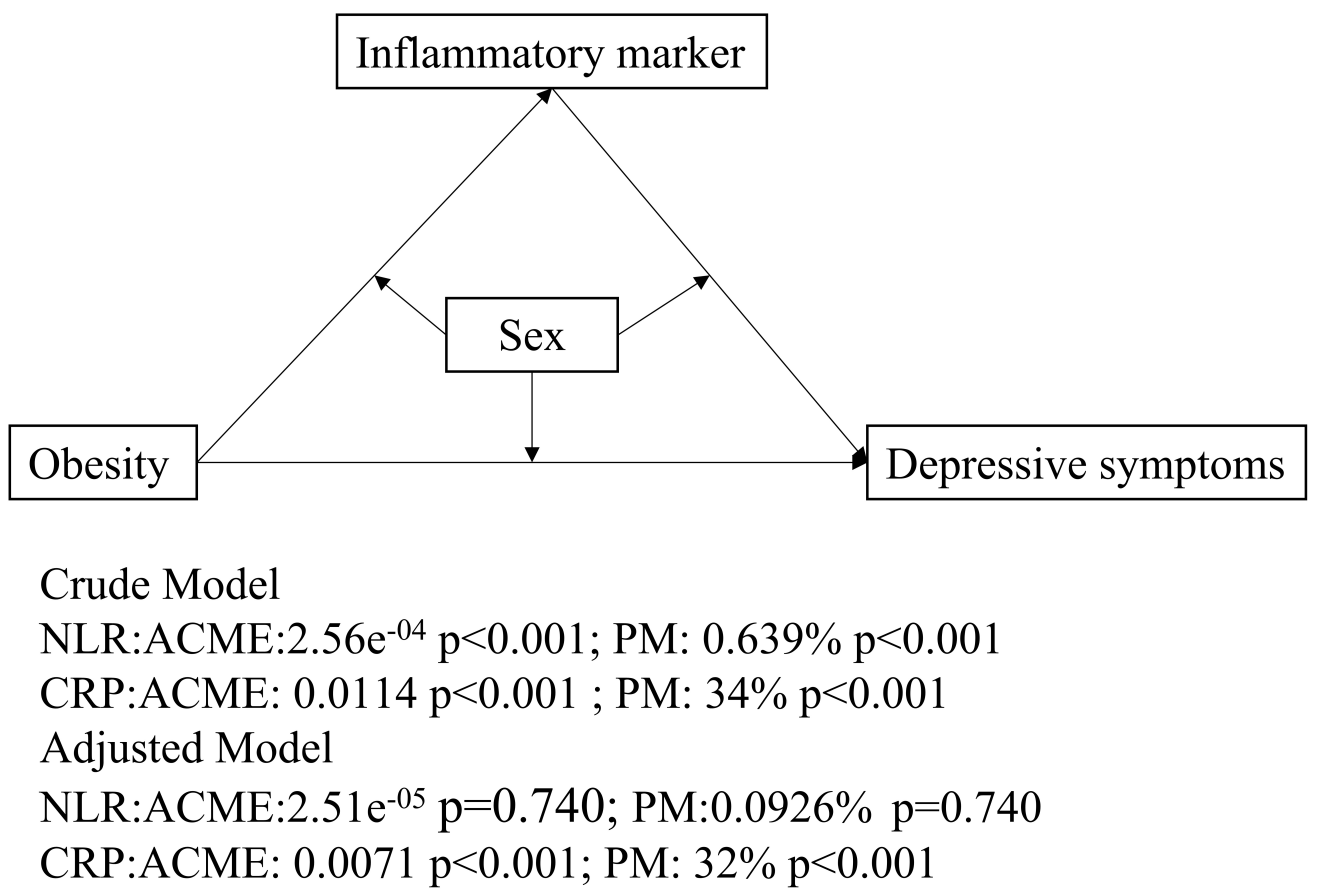
**Mediation models**. The figure illustrates mediation models with obesity as the independent variable, inflammatory markers (NLR or CRP levels) as the mediators, and depressive symptoms as the dependent variable. ACME represents the average causal mediation effects (i.e., the indirect effect), while PM refers to the proportion of the total effect that is mediated by the inflammatory markers. Abbreviations: PM, proportion mediated.

As shown in Table 3 and Fig. 3, both NLR and CRP exhibited mediation effects in the unadjusted model. For NLR, the proportion mediated (PM) was 0.693%, with average causal mediation effects (ACME) of 2.56 × 10^-4^ [95% CI = 1.05 × 10^-4^, 4.70 × 10^-4^], *p* < 0.001. For CRP, the PM was 34% and the ACME was 1.14 × 10^-2^ [95% CI = 0.86 × 10^-2^, 1.41 × 10^-2^], *p* < 0.001. After adjusting for all covariates, the mediation effect for NLR decreased to a PM of 0.0926%, with an ACME of 2.51 × 10^-5^ [95% CI = –1.23 × 10^-4^, 1.90 × 10^-4^], *p* = 0.740. In contrast, CRP continued to demonstrate a significant mediation effect, with a PM of 32% and an ACME of 0.71 × 10^-2^ [95% CI = 0.42 × 10^-2^, 0.98 × 10^-2^], *p* < 0.001. The effect size for the mediation of CRP between obesity and depressive symptoms was moderate, with a proportion mediated of 32% (Cohen’s d = 0.25), indicating a practical but moderate effect.

**Table 3.** **Associations of NLR and CRP with depressive symptoms (n = 37,538)**.

Variable		Crude β (95% CI)	*p*	Adjusted β (95% CI)	*p*	Effect Size (Cohen’s d)
NLR						
ACME						
	Total	2.56 × 10^-4^ (1.05 × 10^-4^, 4.70 × 10^-4^)	<0.001	2.51 × 10^-5^ (–1.23 × 10^-4^, 1.90 × 10^-4^)	0.740	Cohen’s d = 0.02
	Male	5.63 × 10^-4^ (2.44 × 10^-4^, 9.82 × 10^-4^)	<0.001	9.11 × 10^-5^ (–1.76 × 10^-4^, 3.84 × 10^-4^)	0.510	Cohen’s d = 0.08
	Female	1.27 × 10^-4^ (–5.97 × 10^-5^, 3.77 × 10^-4^)	0.160	5.90 × 10^-5^ (–1.01 × 10^-4^, 2.78 × 10^-4^)	0.480	Cohen’s d = 0.05
ADE						
	Total	3.98 × 10^-2^ (3.42 × 10^-2^, 4.58 × 10^-2^)	<0.001	2.71 × 10^-2^ (2.02 × 10^-2^, 3.37 × 10^-2^)	<0.001	Cohen’s d = 0.23
	Male	2.12 × 10^-2^ (1.37 × 10^-2^, 2.84 × 10^-2^)	<0.001	1.56 × 10^-2^ (6.59 × 10^-3^, 2.44 × 10^-2^)	<0.001	Cohen’s d = 0.20
	Female	5.02 × 10^-2^ (4.06 × 10^-2^, 5.94 × 10^-2^)	<0.001	3.65 × 10^-2^ (2.63 × 10^-2^, 4.69 × 10^-2^)	<0.001	Cohen’s d = 0.34
Total Effect						
	Total	4.00 × 10^-2^ (3.45 × 10^-2^, 4.60 × 10^-2^)	<0.001	2.71 × 10^-2^ (2.02 × 10^-2^, 3.36 × 10^-2^)	<0.001	Cohen’s d = 0.25
	Male	2.18 × 10^-2^ (1.42 × 10^-2^, 2.90 × 10^-2^)	<0.001	1.57 × 10^-2^ (6.72 × 10^-3^, 2.50 × 10^-2^)	<0.001	Cohen’s d = 0.17
	Female	5.04 × 10^-2^ (4.08 × 10^-2^, 5.95 × 10^-2^)	<0.001	3.65 × 10^-2^ (2.63 × 10^-2^, 4.69 × 10^-2^)	<0.001	Cohen’s d = 0.34
Prop. Mediated						
	Total	6.39 × 10^-3^ (2.63 × 10^-3^, 1.18 × 10^-2^)	<0.001	9.26 × 10^-4^ (–4.80 × 10^-3^, 7.12 × 10^-3^)	0.740	Cohen’s d = 0.03
	Male	2.59 × 10^-2^ (1.15 × 10^-2^, 4.88 × 10^-2^)	<0.001	5.79 × 10^-3^ (–1.34 × 10^-2^, 2.95 × 10^-2^)	0.510	Cohen’s d = 0.12
	Female	2.51 × 10^-3^ (–1.17 × 10^-3^, 7.52 × 10^-3^)	0.160	1.61 × 10^-3^ (–2.84 × 10^-3^, 7.86 × 10^-3^)	0.480	Cohen’s d = 0.08
CRP						
ACME						
	Total	1.14 × 10^-2^ (0.86 × 10^-2^, 1.41 × 10^-2^)	<0.001	0.71 × 10^-2^ (0.42 × 10^-2^, 0.98 × 10^-2^)	<0.001	Cohen’s d = 0.20
	Male	0.87 × 10^-2^ (0.56 × 10^-2^, 1.15 × 10^-2^)	<0.001	0.72 × 10^-2^ (0.39 × 10^-2^, 1.03 × 10^-2^)	<0.001	Cohen’s d = 0.26
	Female	0.90 × 10^-2^ (0.41 × 10^-2^, 1.47 × 10^-2^)	<0.001	0.50 × 10^-2^ (0.21 × 10^-3^, 1.02 × 10^-2^)	0.046	Cohen’s d = 0.18
ADE						
	Total	2.26 × 10^-2^ (1.51 × 10^-2^, 3.01 × 10^-2^)	<0.001	1.54 × 10^-2^ (0.72 × 10^-2^, 2.33 × 10^-2^)	<0.001	Cohen’s d = 0.24
	Male	0.89 × 10^-2^ (2.18 × 10^-5^, 1.82 × 10^-2^)	0.050	0.54 × 10^-2^ (–0.49 × 10^-2^, 1.54 × 10^-2^)	0.296	Cohen’s d = 0.08
	Female	3.50 × 10^-2^ (2.31 × 10^-2^, 4.64 × 10^-2^)	<0.001	2.58 × 10^-2^ (1.36 × 10^-2^, 3.8 × 10^-2^)	<0.001	Cohen’s d = 0.33
Total Effect						
	Total	3.40 × 10^-2^ (2.64 × 10^-2^, 4.08 × 10^-2^)	<0.001	2.25 × 10^-2^ (1.50 × 10^-2^, 3.00 × 10^-2^)	<0.001	Cohen’s d = 0.22
	Male	1.76 × 10^-2^ (0.90 × 10^-2^, 2.67 × 10^-2^)	<0.001	1.26 × 10^-2^ (0.27 × 10^-2^, 2.22 × 10^-2^)	<0.001	Cohen’s d = 0.18
	Female	4.37 × 10^-2^ (3.32 × 10^-2^, 5.48 × 10^-2^)	<0.001	3.08 × 10^-2^ (1.98 × 10^-2^, 3.80 × 10^-2^)	<0.001	Cohen’s d = 0.34
	Prop. Mediated	0.34 (0.24, 0.46)	<0.001	0.32 (0.18, 0.54)	<0.001	Cohen’s d = 0.25
	Male	0.49 (0.29, 0.98)	<0.001	0.57 (0.25, 2.12)	<0.001	Cohen’s d = 0.28
	Female	0.21 (0.10, 0.36)	<0.001	0.16 (0.01, 0.37)	0.046	Cohen’s d = 0.18

ACME, average causal mediation effects; ADE, average direct effect. Model1: Crude. Model2: Adjusted: Age, Sex, Race, Alcohol, Smoke, Education, Marital, Exercise level, Comorbid.

In the sex subgroup analysis, the mediating effect of CRP was found to be more pronounced in males and less significant in females, indicating that sex serves as an important moderating factor. This indicates that CRP serves as a more reliable mediator in the association between obesity and depressive symptoms, particularly in males (CRP: PM, 57%; ACME = 0.72 × 10^-2^ [95% CI = 0.39 × 10^-2^, 1.03 × 10^-2^], *p* < 0.001 in males; CRP: PM, 16%; ACME = 0.50 × 10^-2^ [95% CI = 0.21 × 10^-3^, 1.02 × 10^-2^], *p* = 0.046 in females).

## 4. Discussion

The aim of this study was to investigate the relationship between obesity and depressive symptoms in American adults, as well as to explore the potential mediating role of inflammatory markers in this association.

Firstly, the study identified a significant association between obesity and depressive symptoms, with the relationship being stronger in women. The bidirectional link between obesity and depressive symptoms is well documented, as obesity increases the risk of depressive symptoms [23], while individuals with depression have a 70% higher likelihood of developing obesity [13]. Furthermore, sex hormones including testosterone, estrogen, and progesterone could significantly influence the occurrence of depressive symptoms in women [12].

Secondly, the results showed a significant correlation between obesity and NLR and CRP levels. Obesity is commonly considered a condition characterized by low-grade chronic inflammation [5], in which macrophages in adipose tissue, especially in abdominal fat, are activated and release pro-inflammatory markers such as TNF-α, interleukin-6 (IL-6), and CRP [24]. The study has also indicated that obesity is associated with changes in glycoprotein acetylation but not with CRP and NLR [25]. Adipocytes serve not only as energy storage cells but also act as endocrine organs. An excess of nutrients in adipose tissue triggers the release of inflammatory mediators such as TNF-α and IL-6, while simultaneously decreasing the production of adiponectin. This imbalance contributes to a pro-inflammatory environment and induces oxidative stress [26]. Additionally, adipocytes release a variety of adipokines and, in obesity, the enlargement of adipocytes may disrupt the balance of these secretions, potentially triggering or exacerbating inflammation [27]. There may also be sex differences in this relationship, with women potentially being more vulnerable to the effects of inflammation [28]. These sex differences could be explained by several factors, including differences in fat distribution, hormone levels, and immune system responses. One potential explanation for the stronger association between obesity and CRP in women is the difference in fat distribution between sexes. Women typically have a higher percentage of subcutaneous fat, while men tend to accumulate more visceral fat, which is more metabolically active and has a stronger inflammatory profile [29]. Hormones, such as estrogen and testosterone, play a significant role in the regulation of adipose tissue and inflammation. Estrogen, which is predominant in women, has been shown to modulate immune responses, potentially influencing the production of CRP [30]. Sex differences in immune system functioning could also explain the observed variations. Women generally exhibit stronger innate and adaptive immune responses, which could lead to a heightened inflammatory response to obesity and contribute to the stronger link between obesity and CRP [31]. These differences in fat distribution, hormones, and immune responses highlight the complexity of the relationship between obesity and inflammation and suggest that sex-specific mechanisms may contribute to the observed variations. Further research is needed to explore these mechanisms in more depth and to consider how interventions might be tailored based on sex.

This study found that NLR and CRP levels were positively associated with depressive symptoms and this association remained significant even after adjusting for covariates. Previous research has similarly demonstrated a bidirectional relationship between inflammation and depressive symptoms [9], as well as a nonlinear relationship between NLR, platelet-to-lymphocyte ratio (PLR), and depressive symptoms [20]. Chronic inflammatory disorders, including those associated with obesity, diabetes, and cardiovascular diseases, have been associated with a higher likelihood of experiencing depressive symptoms [19]. Inflammation may impact brain function through various mechanisms, including neurotransmitter imbalances, neuronal damage, and impaired neurogenesis, all of which may contribute to depressive symptoms [19]. Additionally, the study suggests that anti-inflammatory drugs, such as non-steroidal anti-inflammatory drugs (NSAIDs), could benefit certain patients with depressive symptoms, further supporting the connection between inflammation and depression [32].

Inflammation is thought to play a crucial role in the comorbidity of obesity and depressive symptoms, as higher levels of inflammatory markers are commonly found in individuals with both conditions [33]. This study found that NLR and CRP levels significantly mediated the association between obesity and depressive symptoms in adults. After adjusting for covariates, the mediation effect of NLR was no longer significant, while the mediating effect of CRP increased in men and decreased in women. While the results indicated statistical significances in many comparisons, the large sample size necessitates careful interpretation. As such, effect sizes were provided for a more comprehensive understanding of the magnitude of the associations.

This study is the first to identify inflammatory markers in the relationship between obesity and depression in an adult population, with the advantage of having a large sample size containing diverse racial groups. We used NLR and CRP levels as markers of inflammation, with the strengths of these indicators being their low cost and ease of use in clinical settings. However, there are important limitations to consider. This study, which employed a cross-sectional design, could not establish causal relationships. Although our results revealed a significant link between obesity, inflammatory markers, and depressive symptoms, the direction of these associations remains uncertain. One key limitation of cross-sectional studies is their ability to capture data at a specific moment in time, which prevents the determination of causal effects. Although we observed that inflammatory markers were elevated in individuals with obesity and depression, it is possible that depression may contribute to the development or worsening of obesity and inflammation, rather than the reverse. Therefore, while these associations are noteworthy, future longitudinal studies are necessary to investigate the causal direction and mechanisms underlying these relationships. The use of the PHQ-9 to assess depressive symptoms has certain limitations. As a self-reported questionnaire, the PHQ-9 cannot fully substitute for professional diagnoses. It measures only the severity of depressive symptoms and does not differentiate between subtypes of depression, such as major depressive disorder or anxiety-related depression. Additionally, the study population consisted of community-based samples rather than clinically diagnosed patients with depressive symptoms, which may limit the generalizability of the results to clinical populations. The wide age range of participants introduces variability in physiological and psychological states across different age groups, which could potentially affect the findings. Moreover, this study utilized only two inflammatory markers (NLR and CRP), which may not fully capture the complexity of the inflammatory state. While we have accounted for a range of confounding factors, it is important to note that genetic factors and early life experiences could play a significant role in the observed associations. For instance, genetic variations may predispose individuals to both obesity and depression, making it difficult to disentangle their independent effects. Additionally, early life experiences such as childhood trauma or socioeconomic disadvantage have been shown to influence both mental and physical health outcomes, further complicating the interpretation of these associations. These factors, which were not directly assessed in this study, may affect the observed relationships between obesity, inflammation, and depression.

Future studies should focus on conducting longitudinal research to explore the causal relationships between obesity, inflammation, and depression. More accurate assessment tools, such as professional diagnostic scales for depressive symptoms, should be utilized. The study population should be expanded to include clinically diagnosed patients with depressive symptoms, ensuring greater generalizability of the findings. A broader range of inflammatory markers should also be incorporated to provide a more comprehensive assessment of the inflammatory state. Additionally, the influence of genetic and psychosocial markers should be carefully considered in future research.

## 5. Conclusion

The results of this study revealed significant pairwise associations between inflammatory markers (NLR and CRP levels), obesity, and depressive symptoms. Following the adjustment for covariates, CRP levels were identified as a significant mediator in the association between obesity and depressive symptoms. Subgroup analyses indicated that the mediating effect of CRP was weaker in women compared with men, suggesting that sex differences may influence the interactions between inflammatory markers, obesity, and depressive symptoms. Future longitudinal studies are necessary to determine whether obese individuals are more susceptible to inflammation-driven depressive symptoms, which could ultimately inform personalized treatment strategies for managing depressive symptoms.

## Availability of Data and Materials

The data used in this study are available on the National Health and Nutrition Examination Survey website: https://wwwn.cdc.gov/nchs/nhanes/Default.aspx.
